# Integrating Wearable Textiles Sensors and IoT for Continuous sEMG Monitoring

**DOI:** 10.3390/s24061834

**Published:** 2024-03-13

**Authors:** Bulcha Belay Etana, Benny Malengier, Janarthanan Krishnamoorthy, Lieva Van Langenhove

**Affiliations:** 1 Department of Materials, Textiles and Chemical Engineering, Ghent University, 9000 Gent, Belgium; benny.malengier@ugent.be (B.M.); lieva.vanlangenhove@ugent.be (L.V.L.); 2 School of Materials Science and Engineering, Jimma Institute of Technology, Jimma University, Jimma 378, Ethiopia; 3 School of Biomedical Engineering, Jimma Institute of Technology, Jimma University, Jimma 378, Ethiopia; jana.jk2006@gmail.com

**Keywords:** sEMG, electrode position, smart wearable, textile sensor, IoT-integrated textile sensor

## Abstract

Surface electromyography is a technique used to measure the electrical activity of muscles. sEMG can be used to assess muscle function in various settings, including clinical, academic/industrial research, and sports medicine. The aim of this study is to develop a wearable textile sensor for continuous sEMG monitoring. Here, we have developed an integrated biomedical monitoring system that records sEMG signals through a textile electrode embroidered within a smart sleeve bandage for telemetric assessment of muscle activities and fatigue. We have taken an “Internet of Things”-based approach to acquire the sEMG, using a Myoware sensor and transmit the signal wirelessly through a WiFi-enabled microcontroller unit (NodeMCU; ESP8266). Using a wireless router as an access point, the data transmitted from ESP8266 was received and routed to the webserver-cum-database (Xampp local server) installed on a mobile phone or PC for processing and visualization. The textile electrode integrated with IoT enabled us to measure sEMG, whose quality is similar to that of conventional methods. To verify the performance of our developed prototype, we compared the sEMG signal recorded from the biceps, triceps, and tibialis muscles, using both the smart textile electrode and the gelled electrode. The root mean square and average rectified values of the sEMG measured using our prototype for the three muscle types were within the range of 1.001 ± 0.091 mV to 1.025 ± 0.060 mV and 0.291 ± 0.00 mV to 0.65 ± 0.09 mV, respectively. Further, we also performed the principal component analysis for a total of 18 features (15 time domain and 3 frequency domain) for the same muscle position signals. On the basis on the hierarchical clustering analysis of the PCA’s score, as well as the one-way MANOVA of the 18 features, we conclude that the differences observed in the data for the different muscle types as well as the electrode types are statistically insignificant.

## 1. Introduction

Surface electromyography (sEMG) is an important technique used to monitor the muscle activities of volunteers or patients in sports, prosthesis control, neuro-muscular diseases etc. [1,2,3]. The sEMG measures the sum of the motor unit action potentials (MUAPs) resulting from the activation of the muscle fibers by the motor neuron [4,5]. Conventionally, the sEMG is measured using two electrodes that are connected to a differential amplifier, which basically subtracts these two signals and amplifies them. The ensuing signal is referenced with respect to a third electrode to measure the sEMG signal as a potential difference (volt) [6]. Generally, in hospitals Ag/AgCl electrodes are used, along with a conductive gel applied at the skin–electrode interface. The gel reduces the impedance and improves the signal to noise ratio (SNR) and other signal characteristics [7]. The main disadvantages of the gelled electrode are its fixed form (rigid circular form), drying of its gel on prolonged usage, lower wearability while performing sports or outdoor activities, and its disposal after a single use. In recent decades, dry electrodes have been proposed to replace wet electrodes on the grounds of their wearability (embedded into fabric and other accessories), reasonably good performance, and sustainability features (such as washability and reusability) [8,9,10,11,12]. Unlike the conventional Ag/AgCl gelled electrodes, the wearable dry electrodes can be designed in different shapes (circular, rectangular patch), and require minimal power for functioning. [7,13]. Factors that reduce impedance at the skin–electrode interface, such as sweat and reduced motion artifact, are given significant consideration when designing and evaluating the wearable dry electrodes. The sEMG varies significantly, even for a single muscle type, when electrodes are placed at different loci of the muscle [13]. With no standard procedure in place, customization of electrode positioning is recommended for both dry and gelled electrodes when studying diverse individuals. One desirable feature of dry electrodes is that, since they are embedded into a fabric or a sleeve, the electrode positions itself to the right location of the muscle of an individual who wears it. In a clinical setting, wearable electrodes are more convenient than wet electrodes for the long -term sEMG monitoring of patients [14,15,16]. Textile-based dry electrodes are non-invasive and are comfortable for wearers [17]. Over the past few decades, wearable textile-based “Internet of Things” devices have been demonstrated to be powerful tools for connecting various medical equipment to built-in sensors. Use of IoT in healthcare enables low-cost monitoring solutions by leveraging existing technologies, such as smartphones and wearable devices [6,18], which allows healthcare professionals to deliver superior health services across remote rural areas [19]. Incorporation of IoT technologies to smart sleeves enable the development of sEMG devices with both analog and digital processing capabilities that can undertake real-time monitoring of the muscle activities. To address the motion artifact observed during sEMG monitoring, many studies have suggested the use of smart or intelligent sleeve prototypes [20,21,22,23,24].

In this work, we have developed:

1. A smart sleeve that has a textile electrode for identifying muscle activation. This sleeve is soft, stretchable, and washable, and can be easily incorporated into clothes.
2. IoT-based methodologies are utilized to assess the smart sleeve’s performance of daily muscle activity recognition (MAR).

The dataset of the sEMG from various physical activities, taken from three different muscle positions, is made available to the public.

## 2. Materials and Methods

The methodological approach of this research has two phases. The first phase deals with the development and integration of embroidered textile electrode materials on bandage sleeve garments. The second phase deals with the measurement of real-time sEMG signals [25] transmitted wirelessly to a local application server.

### 2.1. Development of IoT Setup

The developed device consisted of a NodeMCU, which was the microcontroller, and a Myoware sensor. The integration of the IoT with the myoware sensor and the design of the textile electrode is as follows.

#### 2.1.1. Integration of the IoT Device with the sEMG Sensor

**Figure 1:**
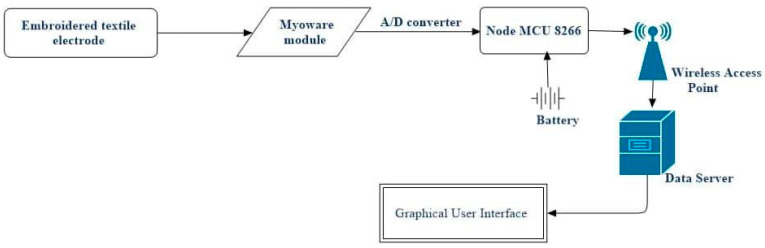
Block diagram of the monitoring device.

**Figure 2:**
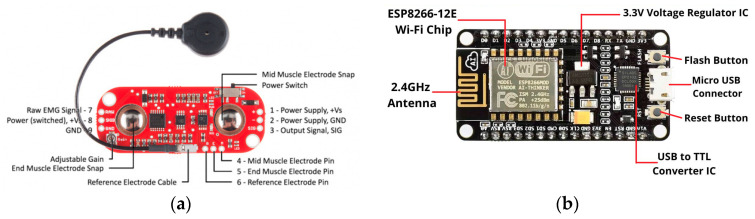
(**a**) Myoware muscle sensor unit, (**b**) node MCU microcontroller unit.

**Figure 3:**
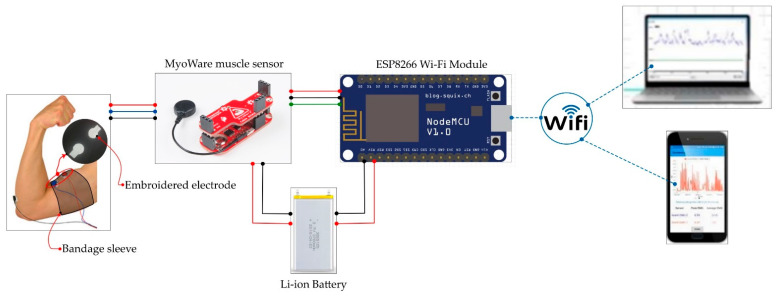
Schematic diagram of the monitoring system.

**Figure 4:**
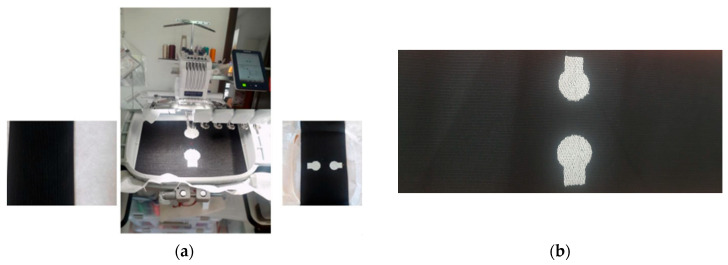
The embroidery process of the textile electrode development, on the textile bandage sleeve of the sEMG monitoring system.

**Figure 5:**
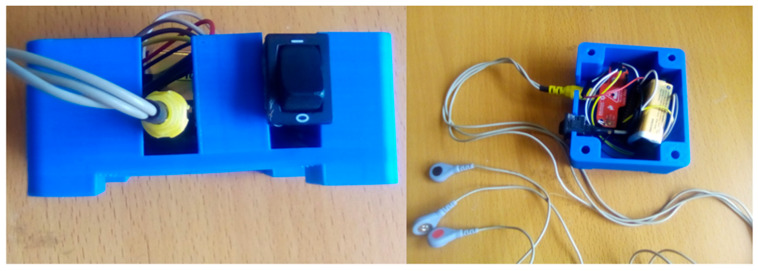
3D-printed final proof-of-concept prototype of the sEMG device, with its housing.

The block diagram of the proposed low-cost portable sEMG textile electrode and associated sensor device is shown in Figure 1. The NodeMCU unit designed by Espresso Systems is equipped with an ESP8266 WiFi module as a wireless component (shown in Figure 2b). The ESP8266 includes a self-contained Wi-Fi networking application, bridging an existing microcontroller with WiFi. On the other hand, the Myoware Muscle Sensor (AT-04-001) developed by Myoware™ (Figure 2a) both senses and processes the sEMG signals. When upstream, it is connected to three electrodes; and when downstream, it is connected to the NodeMCU for signal transmission. The muscular activity is amplified and processed (rectified and integrated) by this sensor on the surface of the skin. The analog signal from the sensor is sent to the Arduino microcontroller with analog-to-digital converter (ADC). The interfacing circuit is depicted in Figure 3, and the design of the textile electrode is shown in Figure 4. Both the NodeMCU 8266 (by Espressif Systems, a public multinational company) and sEMG sensor derive their power from a rechargeable Li-ion polymer battery (3–5 V). The NodeMCU microcontroller, WiFi antenna and battery are placed in a customized 3D-printed rectangular cuboid, as shown in Figure 5. The WiFi antenna connects wirelessly to an access point in the neighborhood, which is typically a mobile phone hotspot. The acquired signal is displayed on a PC or smartphone via the IoT-based monitoring system connected to a locally hosted webserver.

#### 2.1.2. Design of sEMG Measurement Unit

The developed reusable textile-based embroidered electrode was attached to the sleeve, which can be worn directly over the user’s muscle. The overall schematic diagram of the monitoring system is provided in Figure 3. The outer side of the embroidered electrode consists of three snap buttons that will be used to connect with the sEMG sensor. The inner side contains the circular conductive area of the embroidered textrode (diameter 2 cm), which comes into close contact with the muscle. An interconnecting conductive area of dimension, 1 cm × 1 cm, has a snap button stitched to it at one end, and a circular conductive area of the textrode at the other end. The Myoware sensor is attached to the textile electrode through these snap buttons. The inter-electrode distance between the two active electrode patches is 25 mm [25]. The reference electrode is attached to the muscle outside of the sleeve. The prototype of this electrode is shown in Figure 4. Figure 4a presents the process of embroidery electrode on the inner side of the bandage substrate. Figure 4b shows the electrode constituted by the circular region and the interconnecting conductive area. Since the textrodes are used for long-term monitoring of sEMG outside clinical or laboratory settings, their signal quality and reliability must be systematically assessed before they are deployed for any applications [26].

#### 2.1.3. Sensor Interfacing, Data Transmission, and Storage

The acquired data was transmitted by the NodeMCU 8266 microcontroller to a mobile phone or computer via the WiFi protocol with a sampling rate of 60 Hz. Alternatively, the Coolterm (version 2.1.1.1288) software installed on a local system could be used to manage the sEMG signal acquired by the sensor. In our case, we stored the data by using the Xampp application server, which was installed on a mobile phone. From this server, the data could be sent to a cloud-based application server for further processing, using MATLAB (version 2019a) software.

### 2.2. Experimental Set Up

#### Data Acquisition Protocol

This study was approved by the ethics committee of the Jimma Institute of Technology at Jimma University, and also Jimma University’s institutional review board. Before agreeing to participate in the study, participants received information about its purpose and training through the recording procedure. In this study, we aimed to classify three types of muscle groups, namely, biceps, triceps, and tibialis, by using the prototype. Accordingly, the sEMG signal for a healthy person was recorded from three muscles positions, of which two were from the arm and one from the leg. The chosen subject was in the range of somebody who is 28–34-years-of-age. Of the two electrodes with opposite polarity, one was placed on the belly of the muscle and the other was placed away, towards the end of the muscle. The reference was placed far from the target muscle altogether. Both the gel-based electrode (Ag/AgCl 3M, Saint Paul, MN, USA) and the developed textile electrodes were placed with an inter-electrode distance of 25 mm to prevent interference.

Using the tightly fitted sleeve (but comfortable enough for each participant), the signals were measured 5 s after the beginning of the activity. This stabilization period helps to minimize any initial artifacts or fluctuations in the signal that could occur at the outset of the activity. By waiting for the signal to stabilize, researchers can obtain more consistent and valid data for the analysis. Next, the participant was asked to repeat each activity three times, followed by a 2 min rest. The sEMG recording for biceps and triceps muscles were performed in a sitting position. In the tibialis measurement, the subject was asked to walk on the flat floor at normal speed. During the biceps and triceps activities, the participants were asked to flex and extend their forearm with a clenched fist. During the tibialis activities, such as plantar and dorsal flexion, the participants fixed their legs and raised or lowered them within the range of −90 to +90 degrees, depending on the tibialis orientation. The recorded data was refined by removing 5–12%, both at the beginning and the end of the signal. A total of 2 s of the sEMG recording was used in the analysis of each muscle. Subsequently, the 2 s data was segmented into sub data of a 200 ms window period with an overlap of 50 ms. All the experiments and the real-time data collection, for both the textile and gelled electrode, were performed at room temperature.

### 2.3. Principal Component Analysis of Acquired sEMG Signal

A total of nine sEMG signals were recorded from the three muscle types, namely biceps, triceps and tibialis anterior. Of the nine signal dataset, six of were recorded using textile electrodes (one set for each muscle types with repetition), and three signals were recorded using gel electrodes (one set for each muscle type). Time and frequency domain features were extracted from the processed signal [27]. The 15 time domain parameters, such as maximum amplitude value (MAX), mean amplitude value (MEAN), median amplitude value (MED), standard deviation from the mean (SD), variance from the mean (VAR), peak-to-peak distance range (PP), zero crossing (ZC), area under curve (AUC), root mean square (entire segment) (RMS), mean (amplitude) power (MP), mean absolute value (MAV), signal’s energy (EN), waveform length (WL), skewness (SK) and kurtosis (KUR), were obtained. Three frequency domain parameters, namely mean frequency (in power spectrum) (MNF), median frequency (in power spectrum) (MDF) and spectral centroid (SPC), were obtained. The extracted features were subjected to principal component analysis (PCA), in order to identify the significant features that contribute to the variance observed among sEMG signals. The features were normalized using pareito-normal-scaling, $\left(\frac{x_{i}-\mu }{\sigma^{2}}\right)$; with each feature or data point ($x_{i}$) being subtracted from its mean (μ) and divided by its variance, ($\sigma^{2}$). PCA yields the principal components (PC or Eigenvectors) that are used to calculate the score values. The features or data points are projected onto a principal component to obtain the scores. In the ‘score plot’, the scores of one PC (say PC1) are graphically plotted against the scores of another PC (say PC2). In the resulting plot, the data points will, depending on their similarity, segregate into different clusters. In the loading plot, the coefficients of one PC will be plotted against the coefficients of another PC, to identify the significant factors responsible for the clustering observed in the score plot. When the scores and coefficients of two different PCs are plotted together, we obtain a ‘biplot’.

### 2.4. Hierarchical Clustering Analysis

To estimate the significance of the clustering visually observed in PCA plots, we performed the hierarchical clustering based on the scores obtained for the PC1 and PC2 by using the ‘pvclust’ [28] available in the R program [29]. The resulting dendrogram provides *p*-values in %, calculated by either the ‘approximately unbiased’ (AU, shown in red font) or ‘Bootstrap probability’ (BP, shown in green font) approaches. If AU > 95%, then those scores/data are clustered and enclosed within a rectangle.

### 2.5. One-Way MANOVA Analysis

One-way MANOVA (as available in the R package, version 4.3.3) was used to check if there is significant variation among the muscle types or electrode types (independent variable) for the extracted time and frequency domain features (dependent variables). Firstly, we carried out the one-way MANOVA, with muscle type as an independent variable (or a factor with three categories, i.e., triceps, biceps and tibialis) and the 15 TD & 3 FD as the dependent variables. Our observation only contained nine data, and there were twice as many dependent variables (18) meaning, the one-way MANOVA will fail by default. The degree of freedom for the residual is nine (observation or data)—three (groups or muscle type) = six, which is three times less than the dependent variables (18). So we performed the one-way MNOVA analysis with a maximum of five dependent variable (i.e., <6). The resulting Pillai trace statistic was used to obtain an approximate value for the F-statistics. In the analysis, the assumed null hypothesis is that the variance observed for the dependent variables across different groups or factors is not significant. When interpreting the results, if we find the *p* value for the calculated or approximated F-statistics is less than 0.05, we reject the null hypothesis and accept the alternate hypothesis. The alternate hypothesis states that the variance observed for the dependent variables across different groups or factors is significant. In other words, the factors are found to have a significant affect or influence on the dependent variables. Secondly, we also carried out the one-way MANOVA with the electrode type as an independent variable (i.e., a factor with two categories, i.e., Ag/AgCl, textrode) with 15 TD and 3 FD as the dependent variables.

## 3. Results and Discussion

**Figure 6:**
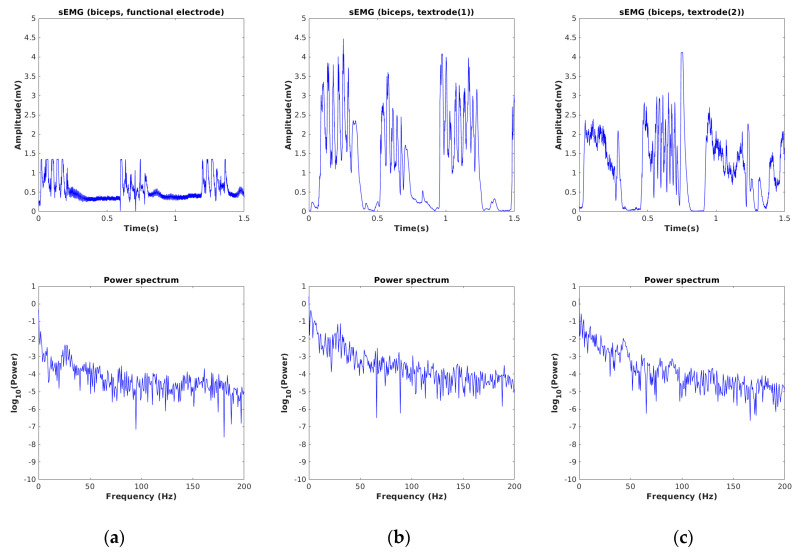
(**a**) sEMG signals recorded from biceps, using gel(functional) electrode, (**b**) conductive hybrid thread-based embroidered electrodes [30], (**c**) the duplicate measurement.

**Figure 7:**
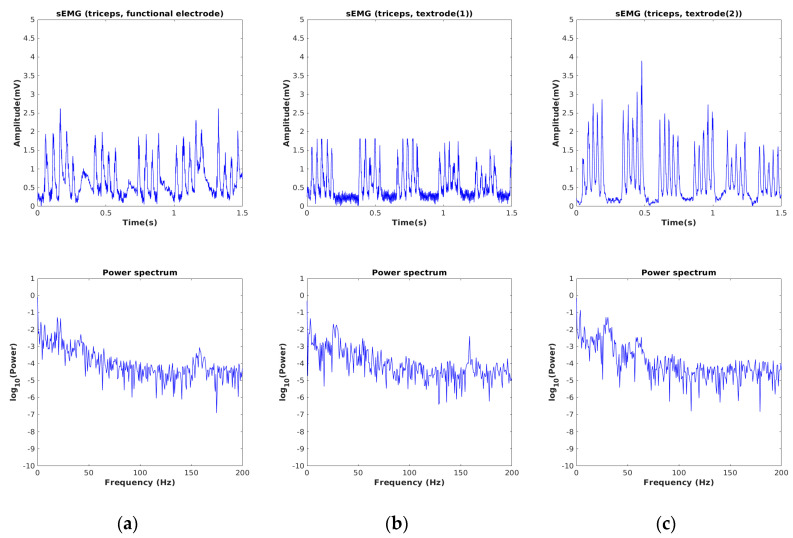
(**a**) sEMG signals recorded from triceps using gelled electrode, (**b**) hybrid thread-based embroidered electrodes (**c**) the duplicate measurement.

**Figure 8:**
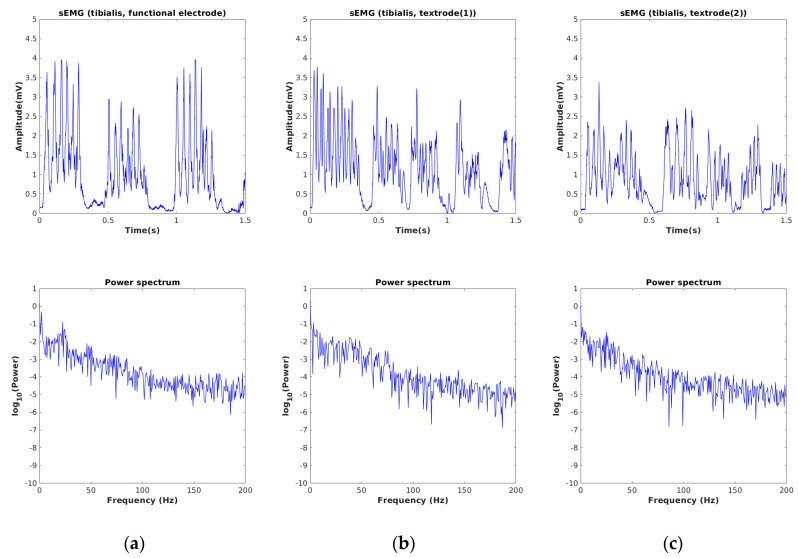
(**a**) sEMG signals recorded from tibialis using gelled (functional) electrode, (**b**) hybrid thread-based embroidered electrodes, (**c**) the duplicate measurement.

The sEMG signals acquired by using our developed device for three different muscle locations of a single subject are depicted in Figure 6, Figure 7 and Figure 8. The sampling frequency was 960 Hz and the data was preprocessed with a digital filter with a lower ($f_{L}$) and upper cutoff frequency ($f_{H}$) of 30 Hz and 450 Hz, respectively. These cutoff frequencies were applied to both the textile electrode and the gelled electrode. To verify the consistency of the textile electrode, we repeated the experiment twice at each muscle location. In each experiment, we calculated the average rectified value (ARV), and root mean square (RMS). The mean and standard deviation (SD) of RMS and ARV, for the textile electrode and the gelled electrode, are presented in Table 1.

In Figure 6, we observe that the denoised signal acquired by using textrode for the biceps appears visually smoother than when the gelled electrode is used. This is also in the SD of RMS values of the textile electrode, which are seen to be close to zero when compared to the gelled electrode. A similar profile was also observed for triceps muscles, as shown in Figure 7. After considering the average RMS and ARV and the signal morphology, we conclude that the embroidered textile electrode is seen to have similar or better signal characteristics, compared to the gelled electrode. The acquired profiles of the tibialis muscle were also similar to those of the other two muscle types (see Figure 8). The power spectrum calculated from the sEMG signal appears similar for all muscle types.

**Figure 9:**
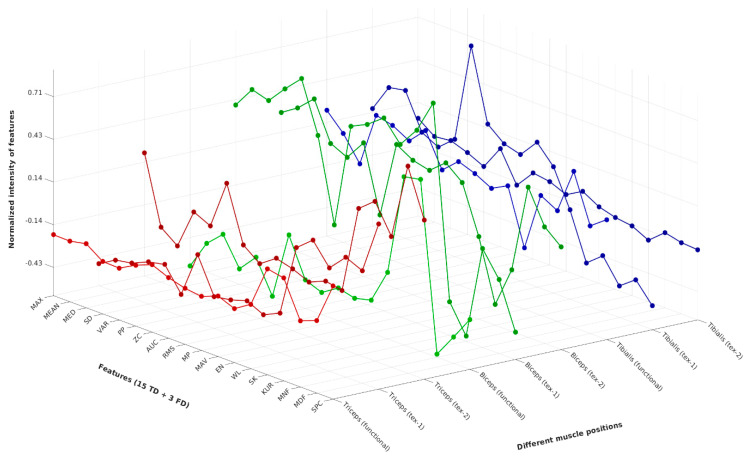
3D plot of the normalized input data used for PCA. The normalized intensities of the pareto-scaled features obtained from the sEMG data of different muscle positions, namely tricep, bicep and tibialis muscles, are shown in the green, red and blue traces, respectively.

**Figure 10:**
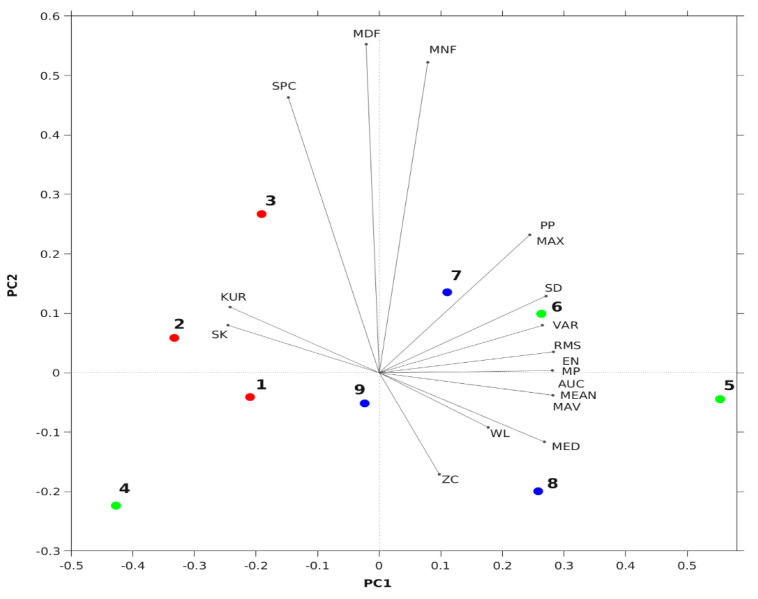
Biplot plot of the sEMG data for textile and gelled (functional) electrodes, compared for tricep (●), bicep (●), and tibialis (●) muscles. The score of the data measured by gelled (functional) and textrode (and its duplicate), which are shown as solid circles; component features corresponding to the first two loading vectors are shown as a line ending with a dot.

Figure 9 shows the 3D plot of the normalized values of the extracted features that were used as inputs for the PCA analysis. The score plot of the PCA exhibits the grouping or clustering of data sets with similar characteristics. In Figure 10, the three label colors, namely the red, green and blue, represent the tricep, bicep and tibialis anterior muscles, respectively. Within the single color code, the sEMG recordings by different electrodes are presented with differing numerical labels. For example, the annotation of the data sets using indices 1, 4 and 7 correspond to the tricep (red), bicep (green) and tibialis (blue) muscles, respectively, and are measured using the gelled electrode. Whereas indices (2, 3), (5, 6) and (8, 9), corresponding to the tricep (red), bicep (green) and tibialis (blue) muscles, respectively, which are measured (with repetition) using the textile electrode.

**Figure 11:**
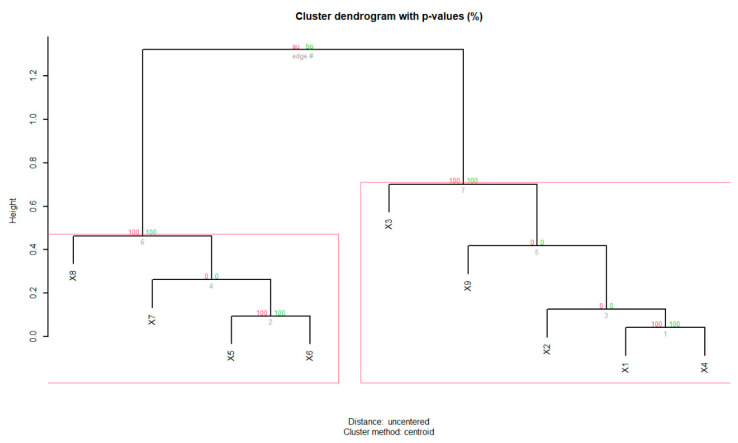
The hierarchical clustering is based on the PC1 & PC2 scores of the nine data: X1, X2, X3 (Triceps), X4, X5, X6 (Biceps), X7, X8, X9 (Tibialis). The analysis has identified two significant clusters bounded by two rectangles, at the significance level of 95% or α = 0.05. We observe that X1–3, which belongs to triceps is, as expected, grouped together (right cluster). But we also observe that the X2 and X9, which belonged to bicep and tibialis data, respectively, are also grouped with triceps. This implies that the variance observed among data (score values) is not very significant and the clustering algorithm could not differentiate each muscle type as separate clusters. Both the AU and BP based *p*-values for each cluster is shown in red and green fonts.

Figure 10 shows the biplot of the loading vectors alongside the score plot of the PCA analysis for 18 features, with 15 TD (time domain) and 3 FD (frequency domain) features derived from the sEMG data. On the basis of the score values, we observe a weak clustering pattern for the tricep, bicep and tibialis muscles. To check for the significance of the observed clusters, we performed hierarchical clustering analysis on the PCA scores that correspond to the PC1 and PC2. Even though two significant clusters were identified (Figure 11), each one contained elements from either two or three different muscle types, which suggests that visually observed variations (Figure 10) among the three muscles were not significant. Furthermore, in referring to the one-way MANOVA performed with muscle type as an independent variable and 18 dependent variables, we obtained a *p*-value of ~0.45, which is greater than 0.05, suggesting that the muscle type did not significantly influence the variance observed for the TD and FD features (Table 2). Likewise, the one-way MANOVA with the electrode type as an independent variable also yielded a *p* value of ~0.73, which is greater than 0.05, suggesting that the electrode types did not significantly influence the observed variance for the TD and FD features (Table 2).

In Figure 10, the bicep scores of the gelled electrode appear separated, as a result of minor displacement of the gelled electrode from its position during flexion and extension exercises. This is one of the reasons why smart sleeves containing textile electrodes are recommended over gelled electrodes, as this will prevent the artifact from being moved, whether due to displacement or slippage effect, during sEMG measurements involving exercises. For continuous long-term monitoring of patients from remote rural areas that uses IoT devices, smart sleeves will be advantageous and more convenient than gelled electrodes, for the aforementioned reasons. The ease in the use of smart sleeves, coupled with the use of IoT devices, significantly improves comfort (soft fabric), adaption (less bulky) and other human factors required for health monitoring devices [31].

A significant part of the spread/variance of scores or clusters (Figure 10) can be explained by referring to the PC1. In particular, parameters such as SK and KUR appear to be higher for triceps, whereas the rest of the features (excluding feature domain parameters: MNF, MDF, SPC) are higher for the triceps and tibialis. The remnant variance present in the data along PC2 can be explained by referring to the frequency domain features (MNF, MDF, SPC). The distinctly separated cluster of triceps from the biceps, as shown in the biplot, can be attributed to the dynamic structural variation (i.e., movement of muscle belly) observed during the experiment, and also to its anatomical differences. A similar observation is reported in a study that compared the maximum voluntary contraction (MVC) of biceps and triceps in normal subjects and patients suffering from spinal cord injury (SCI). It was shown that the MVC of biceps is relatively higher than that of triceps, both for control subjects and SCI cases [32]. This is an indication of the relatively higher muscle activity that the biceps (compared to the triceps) show during flexions and extensions. In another study based on hybrid assistive limb, the sEMG of triceps was shown to be an order of 10 less than the biceps, highlighting the amplitude differences (between these muscle types) observed in the sEMG [33].

A summarized comparison of our work with similar IoT systems for measuring bio potentials that use dry electrodes is provided in Table 3. In general, an IoT system [31] connects (through Bluetooth or a WiFi access point) several sensors to an edge computing device, such as a mobile phone or personal computer. Additionally, it also performs pre-processing, compression or encryption of the data before transmitting it to edge devices. The edge device will in turn transmit the data to a cloud computing system through the internet. Two important factors to consider in IoT design is the acquisition of the source signal (SNR that determines the quality; sampling rate; bit size that determines the quantity) and its reliable transmission (Baud rate) to edge devices. In our study, sEMG acquisition was performed using a single sensor, (i.e., myoware sensor attached to a textile electrode) that was connected to an Aurdino-based IoT system, which in turn reliably captured, processed and transmitted the signal to an edge device, such as mobile phone for visualization that was enabled through a Xampp application server. In its current form, the IoT system can be easily extended to accommodate multiple myoware sensors, and therefore enable simultaneous acquisition of sEMG signals from different parts of the patient’s body.

## 4. Conclusions and Future Work

In this work, we have developed a washable textile electrode for sEMG measurement by embroidering the conductive thread material onto a fabric substrate. The washable electrode was placed in the sleeve and worn on three different muscle types, namely biceps, triceps, and tibialis, to measure the sEMG during various physical activities, such as contraction, relaxation and walking on a flat floor. The experimental results validate that this device is able to detect all the sEMG signals precisely and attain a level of performance comparable to the conventional gelled electrode. Detailed PCA analysis of the features extracted from the sEMG also suggests that the variance of the gelled and textile electrodes is not significant for different muscle types. The current study is limited to a few muscle types and regions, and so future research efforts should be directed towards assessing more muscle position and electrode designs that can be used to monitor the whole human body. Finally, the integration of wearable textile sensors and IoT technology has paved the way for revolutionary advancements in continuous electromyography (EMG) monitoring. IoT seamlessly integrates the flexibility and comfort of wearable textile sensors with robust connectivity. As a result, healthcare professionals and researchers are now equipped with invaluable insights into muscle activity patterns that will facilitate personalized, data-driven interventions, revolutionize approaches to healthcare and drive substantial improvements in patient care.

## Figures and Tables

**Table 1:** Mean and standard deviation (SD) of RMS and ARV of sEMG signal for each muscle, obtained from repeated experiments.

Muscle Group	RMS (mV)	ARV (mV)	Electrode Type
Biceps	1.015 ± 0.001	0.480 ± 0.280	Textile
	1.001 ± 0.091	0.650 ± 0.090	Ag/AgCl
Triceps	1.023 ± 0.001	0.500 ± 0.025	Textile
	1.025 ± 0.060	0.291 ± 0.001	Ag/AgCl
Tibialis	1.010 ± 0.001	0.600 ± 0.110	Textile
	1.016 ± 0.220	0.513 ± 0.270	Ag/AgCl

**Table 2:** The *p*-values obtained for the one-way MANOVA, using either the muscle type or electrode type as an independent variable (factor) and 18 features as dependent variables. Since the observation data is less than the dependent variables, the MNOVA was done in batches. A *p*-value less than 0.05 was considered to be significant.

Independent Variable	Dependent Variables	Pillai Trace Statistics	Aprroximated F Value	*p*-Value
Muscle Types (Tricep, Bicep, Tibialis)	MAX, MEAN, MED, SD, VAR	1.3529	1.2545	0.4068
	PP, ZC, AUC, RMS, MP	1.3141	1.1495	0.4515
	MAV, EN, WL, SK, KUR	1.2655	1.0338	0.5071
	MNF, MDF, SPC	0.7766	1.0579	0.4456
Electrode Types (Ag/AgCl Gelled, Textrode)	MAX, MEAN, MED, SD, VAR	0.4671	0.5259	0.7526
	PP, ZC, AUC ,RMS, MP	0.3326	0.2990	0.8866
	MAV, EN, WL, SK, KUR	0.4848	0.5646	0.7312
	MNF, MDF, SPC	0.3161	0.7703	0.5581

**Table 3:** Comparison of the current study to similar reported studies of on the usage of the dry electrodes when integrated with the IoT system.

Application	Materials	Method of Application and Limitation	Method of Evaluation	Reference
Smart textile for measuring trunk orientation	Embroidered-based conductive threads, HC-40 and C-70	Development and integration of sensor materials into textile garment, when full integration of IoT was not implemented	Electrode comparison	[33]
Electromyography (sEMG) recording	Graphene based textile electrode	Ozturk et al. utilized dip coating and sewing techniques to integrate electrodes into the bandage sleeve during the development of a sEMG device. However, when dip coating and sewing methods are compared to embroidery electrodes, the latter are often preferred for their comfort, durability, flexibility, customization options, and potential for better signal quality, especially when integrated into the IoT system	Performance evaluation	[34]
Electrocardiography (ECG)	Conductive fabric-based wearable device integrated with the IoT,	Sewn electrodes may be prone to loose contact with the skin during movement, leading to signal quality issues. In contrast, embroidered electrodes are seamlessly integrated into the fabric, reducing the risk of contact loss and improving signal quality, especially when used in IoT applications for continuous monitoring. The flexibility and customization options of embroidery also make it a preferred choice for wearable technology that requires reliable and high-quality sensor data.	Electrode comparison	[35]
sEMG	Conductive Hybrid threads	Textrode embroidered onto the sleeve and integrated with the IoT system	Muscle type comparison	Current work

## Data Availability

The datasets used and/or analyzed by this paper can be obtained from the corresponding author upon reasonable request.
